# The therapeutic relationship in EMDR therapy—A survey

**DOI:** 10.3389/fpsyg.2025.1519665

**Published:** 2025-03-17

**Authors:** Michael Hase, Karl Heinz Brisch, Roger M. Solomon, Adrian Hase

**Affiliations:** ^1^EMDR Center, Lüneburg, Germany; ^2^Institute for Early Life Care, Paracelsus Medical University, Salzburg, Austria; ^3^Dr. von Hauner Children’s Hospital, University of Munich, Munich, Germany; ^4^EMDR Institute, Watsonville, CA, United States; ^5^Section of Medicine, University of Fribourg, Fribourg, Switzerland

**Keywords:** EMDR therapy, adaptive information processing, therapeutic relationship, attachment theory, training, research

## Abstract

The history of EMDR therapy goes back to 1987, when it was introduced as EMD, a novel treatment for PTSD by Francine Shapiro. Over the course of time EMD developed into the comprehensive therapy approach named EMDR therapy. The development of the Adaptive Information Processing (AIP) Model, the model of pathogenesis and change of EMDR therapy, was a milestone in this development from technique to psychotherapy approach. Lately a description of the therapeutic relationship in EMDR therapy has been proposed based on attachment theory. The therapeutic relationship has been described as a core element of EMDR Therapy, and seems to be related to the structure of EMDR Therapy. An internet-based survey of EMDR therapists in several waves was used to evaluate whether EMDR therapists support the above mentioned description of the therapeutic relationship in EMDR therapy. The self-experience of the EMDR therapists in EMDR therapy as elicited in the survey seems to support the description of the therapeutic relationship in EMDR therapy. Even if the survey was only conducted with EMDR therapists, thus limiting the informative value on the patient population in general, it offers valuable insights into the therapeutic relationship in EMDR Therapy. Implications for treatment, training and research will be discussed.

## Introduction

Eye Movement Desensitization and Reprocessing therapy (EMDR) consists of a structured set of treatment plans and procedures based on the Adaptive Information Processing (AIP) model (Solomon and Shapiro, 2008). EMDR was introduced as EMD in 1987 (Shapiro, 1989) as a treatment for PTSD and was developed into the comprehensive therapy approach named EMDR therapy over the following years. Shapiro intended EMDR therapy to be compatible with all major orientations of psychotherapy.

The theory currently used to guide EMDR Therapy treatment effects is called the Adaptive Information Processing (AIP) model. The AIP model was developed to explain the rapid change toward positive resolution that can be seen in the EMDR memory reprocessing (Shapiro, 1995, 2001, 2018). The AIP model assumes “the physiological systems of the brain that attend to the assimilation of experience are no different from other systems” and “The movement toward health and balance is sustained unless there is a block or repeated traumatization” (Shapiro, 2002, p. 8). The term information as used in EMDR therapy refers to affect, cognition, sensory, somatosensory or other internal or external input as perceived at the time of the event leading to memory formation. In EMDR therapy, it is presumed that the activity of the adaptive information processing system leads to the integration of dysfunctionally encoded information toward functional encoding and adaptive state of memory, in consequence contributing to reduction in distress and/or negative emotions. The AIP system may be hindered or blocked by trauma, other severe stress, or other like the influence of psychoactive drugs in consequence leading to formation of the maldaptively stored memory, which is assumed to be foundational for psychopathology. Shapiro described the adaptive information processing system an ‘innate’ system. But even if we assume that the adaptive information processing system is active from birth, we should take into account that every system like speech, movement, e.g., needs to develop. A developmental perspective of the adaptive information processing system is still missing in EMDR therapy.

In contrast to a common, but limited perception of EMDR therapy that the AIP model is only a model of inadequately processed memories, it also involves adaptive information, or to say of resilience (Solomon and Shapiro, 2012). Reprocessing procedures promote the linking in of positive, adaptive information into the maladaptively stored information, promoting adaptive resolution and integration into the wider memory network.

Shapiro stressed the fact that the client would need sufficient positive memory networks, which are present and accessible, for successful memory reprocessing (Shapiro and Laliotis, 2017). When there is a lack of adaptive information or integrative capacity, it seems straightforward that we need to install adaptive information prior to reprocessing. Common procedure are Resource Development and Installation (RDI) (Korn and Leeds, 2002) and the Instant Resource Installation (IRI) procedure (Hase, 2021).

Regarding posttraumatic stress disorder (PTSD), memory deficits, negative beliefs concerning the self, avoidance or suppression of memories, and negative interpretation of memory symptoms could explain impairment of access to adaptive memory networks (Brewin, 2011). The mere activation of malaptively stored memory could initiate a stress response altering the brains capacity to access adaptive memory or resulting in the loss of dual attention (Shapiro, 2018; Brewin, 2018). But it is not only PTSD, which can be the cause behind impaired access to adaptive memory. Survivors of early neglect show impaired capacities to tolerate and integrate moments of shared positive interpersonal experience. Into positive emotional states and positive self-concepts. They tend to make use of overt or covert avoidance strategies and minimization responses to avoid the discomfort, anxiety, or confusion connected to positive experiences. This defensive avoidance significantly contributes to symptom maintenance. EMDR therapy includes a preparation and stabilization Phase (Phase 2 of EMDR therapy) and involves providing interventions to enable to meet readiness criteria for EMDR memory work, such as the ‚Positive Affect Tolerance and Integration Protocol (Leeds, 2022). In general EMDR therapy preparation and resourcing interventions aim to expand the brain‘s integrative capacity and strengthen adaptive memory networks (Shapiro and Laliotis, 2017, p. 10, 18). Further, it is important to build a strong enough therapeutic relationship in preparation for the upcoming reprocessing of maldaptively stored information.

Shapiro was even more precise regarding the importance of the ability to access adaptive memory networks for reprocessing to occur. The 2017 edition of the EMDR Institute Basic Training Manual, and the current 2023 edition, emphasize the necessity of present and accessible adaptive memory networks and the importance of the therapeutic relationship as an source of adaptive information. “Adaptive memory networks need to be present and accessible for reprocessing to occur. Therapeutic relationship is part of an adaptive memory network” (Shapiro and Laliotis, 2017, p. 13).

Interestingly Shapiro (2007) discussed the therapeutic relationship in her textbook (2018), even more explicitly than in the EMDR Basic Training Manual, and advised that the therapist behavior be “optimally interactive (p. 76),” but refrained from describing the therapeutic relationship in EMDR therapy in detail or on how to establish a secure therapeutic relationship prior to memory reprocessing in detail. As the therapeutic relationship is of great importance regarding the outcome in psychotherapy (Orlinsky et al., 1994) this issue needs to be addressed. Hase and Brisch (2022) provided a description of the therapeutic relationship in EMDR therapy based on attachment theory (Bowlby, 2005). Predictability of the attachment figure is a good basis for the development of the infant. One could assume, that the predictability of therapist behavior in the manualized procedures of EMDR therapy contributes to the development of the therapeutic relationship in EMDR therapy.

According to Brisch (2002) the development of attachment between the infant and caregiver is related to the sensitivity of the caregiver. The caregiver with the most sensitive properties will become the primary attachment figure of the infant. Sensitivity facilitates the development of attachment. How could one define sensitivity? Brisch (2002) outlined that sensitivity shows in speech, rhythm, eye contact and touch. Most important the caregiver has to be able to perceive the infants’ signals while not misinterpreting them, and react immediately and appropriately. This is only possible if the caregiver is emotionally available for the needs, affects and signals of the infant. In EMDR therapy as therapists we offer speech, but even more important, rhythm. We keep eye contact, while being not intrusive. Sometimes we offer touch. But most important, we perceive our clients’ signal, being aware not to intrude, while reacting promptly and appropriately, which will certainly facilitate the development of a secure therapeutic relationship. The therapeutic relationship not only results from the above-mentioned behaviors, but also develops through procedures and protocols within EMDR therapy. Given the therapeutic relational factors described above, EMDR can be described as a ‚sensitive‘psychotherapy approach. Table 1 shows EMDR therapy actions in relation to sensitive behavior.

**Table 1:** Sensitivity and EMDR therapy.

Sensitive behavior	EMDR therapy
Speech	Help to elicit cognition; verbal support during set
Rhythm	Bilateral stimulation; timing of sets and breaks
Eye Contact	Aware of facial expression; Eye Movements
Touch	Tactile Stimulation
Perception of client	Awareness during BLS
Not misinterpreting	Refraining from comments; ‚stay out of the way‘
Prompt and appropriate	Keep client in stimulation and react promptly to affective stress of client

These actions can explain at least to a great extent the specific therapeutic relationship in EMDR therapy, which often develops rapidly and provides a safe shared space, allowing our clients to reprocess, to grow and to get past their past. This article describes the results of an internet-based survey eliciting information on the self-experience of therapist during their training in EMDR therapy in order to evaluate the sensitivity factors facilitating the development of the therapeutic relationship in EMDR therapy.

EMDR therapy basic training as offered by the EMDR Institute Inc. or following the standards of EMDR Europe (website) consists of 52 h. These are divided into 22 h of theory and 20 h of a supervised practical self-experience, the so-called practicum, followed by 10 h of clinical case consultation. The 42 h of theory and practicum are mostly offered in two blocks of 3 days each. Within the practicum the attendees are advised to rotate through the roles of client, therapist and observer. In the role of client, the attendee is asked to bring up his own distressing memories which will be reprocessed with the help of the therapist. The attendee should experience the therapeutic action of EMDR therapy procedures, mostly the standard EMDR protocol, including the reprocessing on their own memories. This self-experience is an important part of the EMDR therapy basic training. For successful completion of the basic training an attendee is expected to be at least in the role of client and therapist once on each block. One can assume that after completion of the 42 h of theory and practicum each attendee has provided self-experience as a client in EMDR therapy memory reprocessing at least two times. In addition, the attendee has been in the role of client regarding diagnostic and preparatory procedures in the practicum. This is a minimum of self-experience in EMDR therapy basic training. Attendees are encouraged to engage in additional self-experience, but this not mandatory. As a result, the therapist practicing EMDR therapy after attending an EMDR therapy basic training could be questioned regarding their experience.

## Methods

We made up a questionnaire describing core elements of the therapeutic relationship according to the above-mentioned sensitive factors in EMDR therapy. The EMDR therapist was asked to rate if they would disagree or agree on a five point scale. Responses were scored on a 5-point scale (1 = *Strongly disagree*, 2 = *Disagree*, 3 = *Neutral*, 4 = *Agree*, 5 = *Strongly Agree*) regarding a statement describing their experience as client in EMDR therapy. In addition, we asked for the number of EMDR sessions in the role of client and some demographic information as age and gender. The questionnaire was distributed in a pilot run and two waves in a modified Delphi-approach (Niederberger and Spranger, 2020). The results of each wave were used as feedback in order to modify the questionnaire. The results of wave one were also analyzed statistically contributing to another modification which led to the final version called BET 5.2. BET stands for „Beziehungserleben in der EMDR Therapie “which could be translated as „Relational Experience in EMDR therapy“. The BET 5.2 was tested in the second wave of the survey. Even if the survey was only conducted with EMDR therapists, thus limiting the informative value on the patient population in general, it could offer valuable insights into the therapeutic relationship in EMDR therapy. One has to keep in mind, that psychotherapist are of course suffering from mental health problems. In a study on clinical, counseling, and school-psychology faculty and trainees overall rates of mental disorders were similar to or greater than those observed in the general population (Victor et al., 2022). With more than 80% of respondents (*n* = 1,395 of 1,692) reporting a lifetime history of mental-health difficulties, and nearly half (48%) reporting a diagnosed mental disorder the burden of mental-health problems seems high. Among the reported concerns the most common were depression, generalized anxiety disorder, and suicidal thoughts or behaviors. A recent study suggested a better overall mental health profile for Austrian psychotherapists compared to the population in general (Schaffler et al., 2024). Still quite a number of psychotherapists in this study exhibited scores beyond the cut-off for clinically significant mental disorders like depression, anxiety or insomnia. A survey among UK-based psychologist revealed, that Two-thirds of the participants had experienced mental health problems themselves (Tay et al., 2018). Disclosing and help-seeking were impaired by concerns about negative consequences for self and career. The reported rates of mental-health problems seem to make the psychotherapists in our sample a reasonably representative group, though most likely not representing the severly disordered, multiply traumatized client.

A preliminary version of the questionnaire was distributed in a pilot trial as an online survey (implemented via surveymonkey.com) to 16 experienced therapists who had participated in an online seminar on the therapeutic relationship in EMDR therapy. This version consisted of 13 descriptive items plus the additional demographic items. Feedback from this group of experts led to a questionnaire of 12 items describing the experience in the role of client. This questionnaire was then used in the following two survey waves to optimize the questionnaire using statistical methods.

### Participants

The first author used an e-mail list of EMDR therapists. The minimum requirement to become member of this list is completion of the first part of the EMDR Therapy basic training. There were 1,348 therapists registered on the email list. Out of the 1,348, 118 responded in the first wave. The first wave of the survey comprised 118 individuals (82.2% female) who had experienced between one and five EMDR sessions (*M* = 2.28, *SD* = 1.15). Participants’ age ranged from 26 to 80 years, with the majority (61.2%) being between 40 and 59, 32.8% between 60 and 79, and 6.0% between 20 and 39 years old.

### Materials

We tested in the beginning 12 descriptive items to measure the experience of the therapeutic relationship in EMDR therapy. These items were developed by a senior EMDR trainer with 25 years of experience in EMDR therapy and had been tested in a pilot study. Responses were scored on a 5-point scale (1 = *Strongly disagree*, 2 = *Disagree*, 3 = *Neutral*, 4 = *Agree*, 5 = *Strongly Agree*). Table 1 presents item contents and descriptive statistics. According to results in the trial run and first wave the number of items was reduced to nine.

### Procedure

After the trial run the preliminary descriptive items were distributed in an online survey (implemented via surveymonkey.com) to the participants via an email list. Participants at first responded to 12 core survey items in the first wave. The statistical analysis suggested reducing the number of items to nine. In the second wave of the survey, the nine descriptive items and other information about the number of EMDR sessions experienced and demographics were acquired. The second wave of the survey was sent out via the same email list and 149 individuals responded. Table 2 shows the item content and descriptive statistics.

**Table 2:** Item contents and descriptive statistics.

Item	Content	*M*	*SD*	*Min*	*Max*
1	“The therapist controls the process through my eye movements, touch, or sounds. I find that pleasant in the relationship.”	4.12	0.76	2	5
2	“I experience the neutral feedback from the therapist during an EMDR session as pleasant.”	3.90	0.77	2	5
3	“That the therapist reacts immediately to changes within the EMDR session is agreeable.”	4.12	0.84	1	5
4	“The repetitive questions or statements (e.g., ‘What do you get now’, ‘Follow my fingers …’) by the therapist induce a sense of safety.”	4.13	0.80	2	5
5	“During an EMDR session with stimulation by eye movements, touch or tones I feel myself as being in the awareness of the therapist.”	4.19	0.89	0	5
6	“The structure in the EMDR reprocessing (working through a memory) given by the therapist (briefly talking about the memory - followed by eye movements, e.g., – short feedback – eye movements, e.g., - …) induces a sense of safety.”	4.22	0.68	2	5
7	“I developed confidence in EMDR therapy faster than expected.”	4.25	0.79	2	5
8	“Over the course of EMDR therapy, the relationship with my therapist deteriorated.”	4.33	0.90	1	5
9	“During an EMDR session patient and therapist are working together in collaborative manner.”	4.42	0.63	2	5
Scale		4.21	0.45	0	5

### Statistical analysis

All statistical analyses were conducted in RStudio, version 2023.12.1. We used the fa.parallel() function of the *psych* package to conduct exploratory parallel analysis to determine the recommended number of factors and create scree plots (Revelle, 2024). We then used the base R function fa() with the maximum-likelihood factor analysis factoring method, varimax rotation, and a maximum of 100 bootstrap iterations to assess the underlying factor structure and corresponding fit indices in an exploratory factor analysis specifying the previously recommended number of factors. We visualized the factor structure of the recommended model in factor loading plots using the fa.diagram() function of the *psych* package. We applied a significance level of *α* = 0.05.

### Results

**Figure 1:**
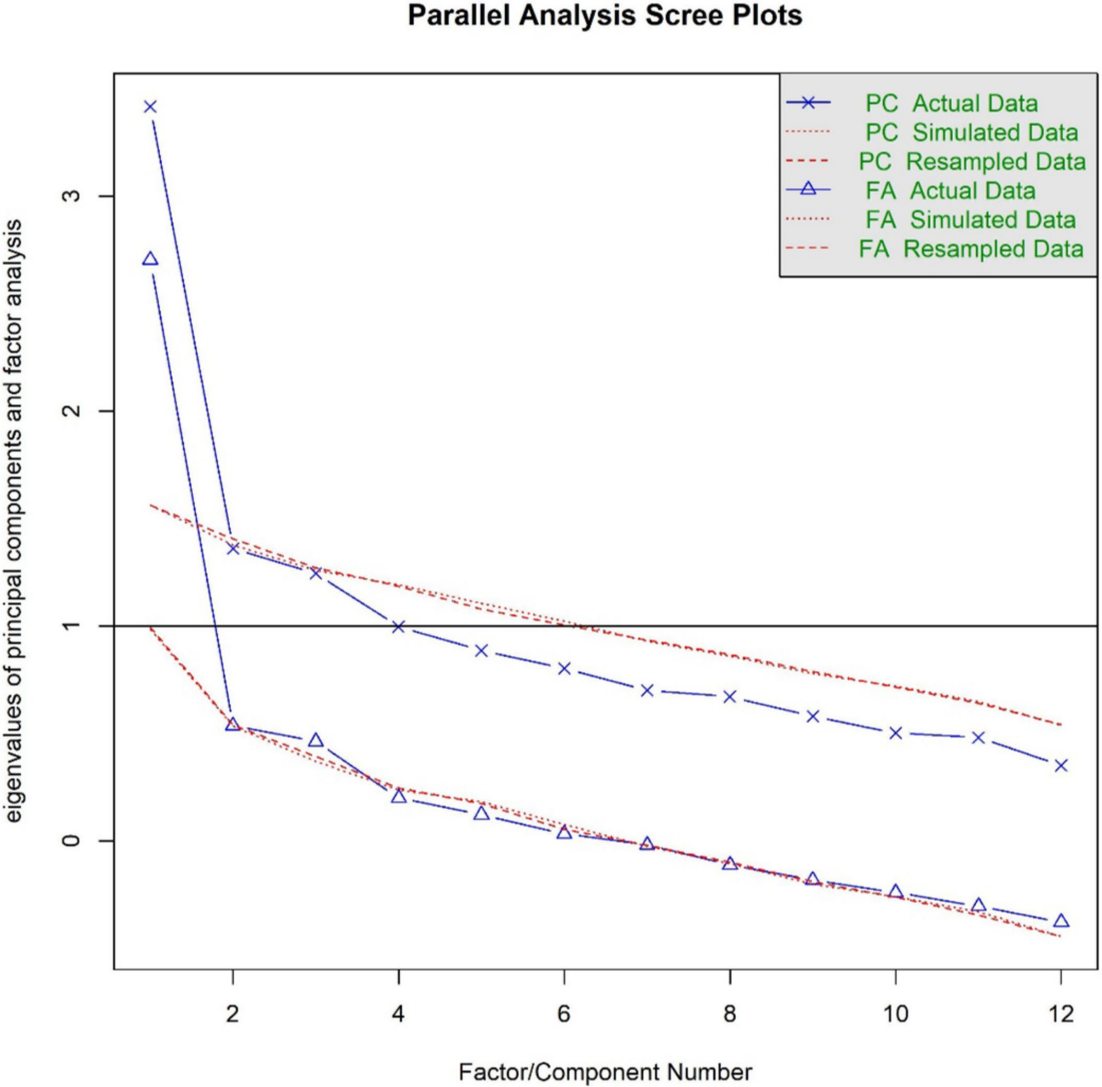
Parallel analysis scree plots (wave one).

**Figure 2:**
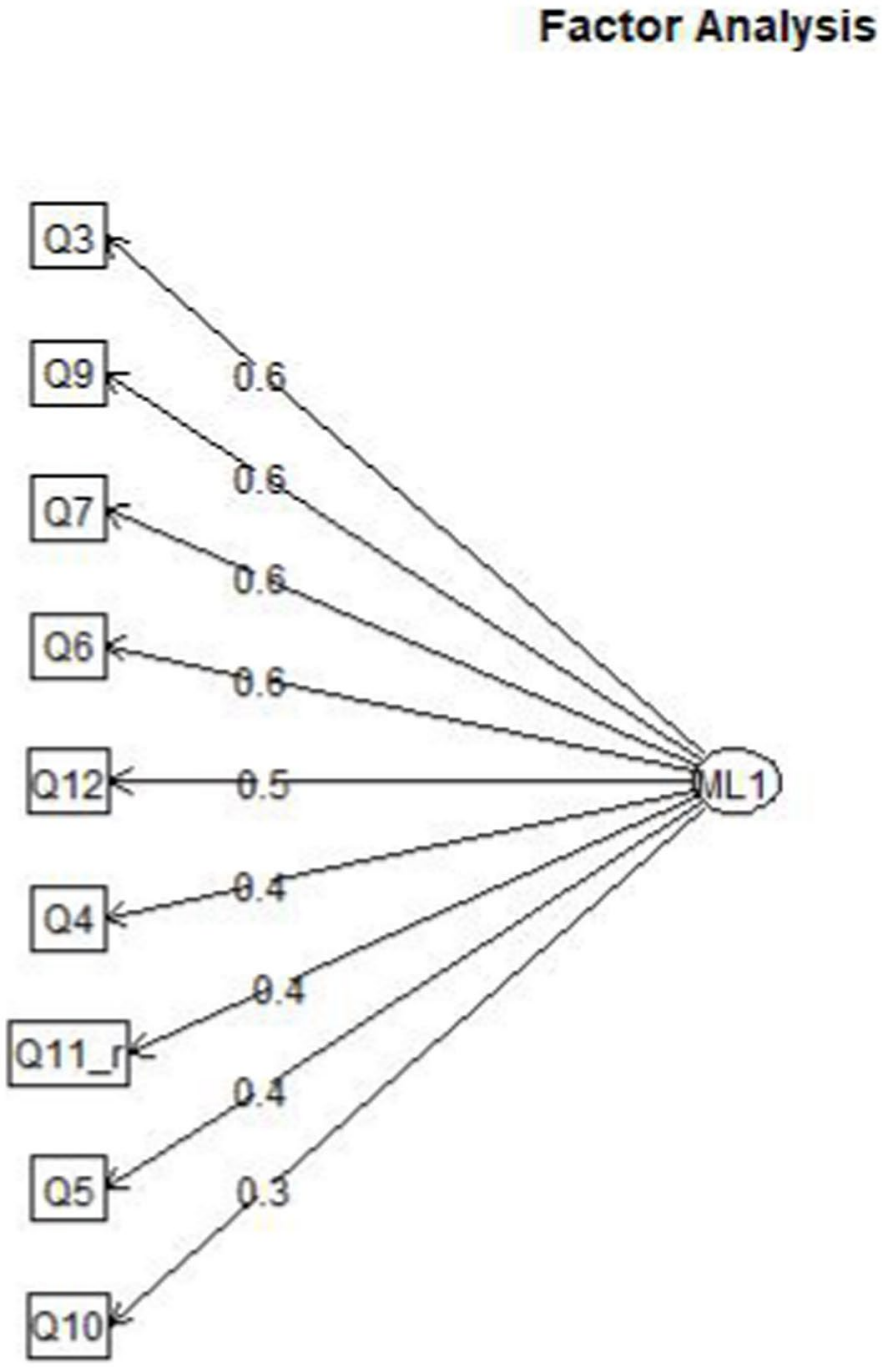
Factor loading plot (study 1).

Figure 1 presents scree plots from the parallel analysis, which recommended a one-factor solution. The exploratory factor analysis for the one-factor solution showed a satisfactory fit for the recommended model, TLI = 0.912, RMSEA = 0.054, 95% CI: < 0.001, 0.097. The factor score adequacy was acceptable with a regression score-factor correlation of 0.88 and a multiple *R*^2^ of scores with factors of 0.77. Figure 2 presents the factor loading plot for the recommended one-factor solution.

### Wave two

The aim of wave two was to validate the one-factor solution identified in wave one. We hypothesized that the one-factor model would be confirmed in an independent sample and exhibit acceptable psychometric properties.

### Method and participants

To validate the factor structure found in the first study, the modified nine core item version of the questionnaire was sent out via the same email list. A total of 149 individuals (81.0% female) participated in the second wave. Participants’ age ranged from 26 to 80 years, with the majority (51.7%) being between 40 and 59, 41.6% between 60 and 79, and 6.7% between 20 and 39 years old. The experience in the role of client with EMDR therapy was rather balanced, with most participants having experienced a moderate number of sessions (38.3% between 5 and 12 sessions, 30.2% between 0 and 4, 20.1% between 13 and 24, 8.7% between 25 and 50, and 2.7% more than 50 sessions).

#### Procedure

The questionnaire data collection process was the same as in wave one. There was no missing data on any of the questionnaire items.

#### Material

We used the revised nine core item questionnaire. There was no missing data.

### Results

A total of 77.19% agreed or strongly agreed to item 1 “The therapist controls the process through my eye movements, touch, or sounds. I find that pleasant in the relationship.”

A total of 76.51% agreed or strongly agreed to item 2 “I experience the neutral feedback from the therapist during an EMDR session as pleasant.”

A total of 87.25% agreed or strongly agreed to item 3 “That the therapist reacts immediately to changes within the EMDR session is agreeable.”

A total of 81.88% agreed or strongly agreed to item 4 “The repetitive questions or statements (e.g., ‘What do you get now?,’ ‘Follow my fingers …’) by the therapist induce a sense of safety.”

A total of 87.25% agreed or strongly agreed to item 5 “During an EMDR session with stimulation by eye movements, touch or tones I feel myself as being in the awareness of the therapist.

A total of 85.93% agreed or strongly agreed to item 6 “The structure in the EMDR reprocessing (working through a memory) given by the therapist (briefly talking about the memory - followed by eye movements, e.g., – short feedback – eye movements, e.g., - …) induces a sense of safety” 6.

A total of 81.11% agreed or strongly agreed to item 7 “I developed confidence in EMDR therapy faster than expected.”

86% disagreed or strongly disagreed to Item 8 “Over the course of EMDR therapy, the relationship with my therapist deteriorated.” Item scoring was reversed in the statistical analysis. This item checks if the person carefully read and understood the proposed item.

A total of 91.28% agreed or strongly agreed to item 9 “During an EMDR session patient and therapist are working together in collaborative manner.”

Summarizing the answers we found very good support for a concept of a collaborative therapeutic relationship which develops faster than clients expected and seems robust (items 7, 8, 9). In addition we found very good support for the core items describing sensitive therapist behavior (items 1–6).

Table 3 presents the number of answers on the five point scale in %.

**Table 3:** Number of answers on the five point scale in % related to question/item.

Item	Strongly disagree	Disagree	Neutral	Agree	Strongly agree
1	0.00	2.68	20.81	56.38	20.81
2	0.67	2.68	20.13	52.35	24.16
3	0.00	0.00	12.75	48.32	38.93
4	0.00	3.36	14.77	52.35	29.53
5	0.00	4.03	9.40	46.31	40.94
6	0.00	0.67	14.09	40.27	45.64
7	0.67	5.37	12.75	36.91	44.30
8	52.35	33.65	8.72	3.36	2.01
9	1.34	0.67	6.71	40.94	50.34

### Statistical analysis

The next was to analyze the data. Table 4 presents descriptive statistics for the individual items as well as the entire scale, which showed good internal consistency, *α* = 0.84, 95% CI: (0.80, 0.88).

**Table 4:** Scale descriptives.

Item	*M*	*SD*	*Min.*	*Max.*
1	3.94	0.72	2	5
2	3.97	0.78	1	5
3	4.26	0.67	3	5
4	4.08	0.76	2	5
5	4.24	0.78	2	5
6	4.31	0.73	2	5
7	4.19	0.90	1	5
8	4.31	0.91	1	5
9	4.38	0.76	1	5
Scale	4.19	0.52	0	5

We used the cfa()function of the *lavaan* R package (Rosseel, 2012) to perform confirmatory factor analysis on the factor solution identified in the first wave data. We calculated Cronbach’s alpha and corresponding confidence interval values using the *psych* package (Revelle, 2024). We visualized the confirmatory factor analysis results using the *semPlot* package (Epskamp, 2022).

## Results

**Figure 3:**
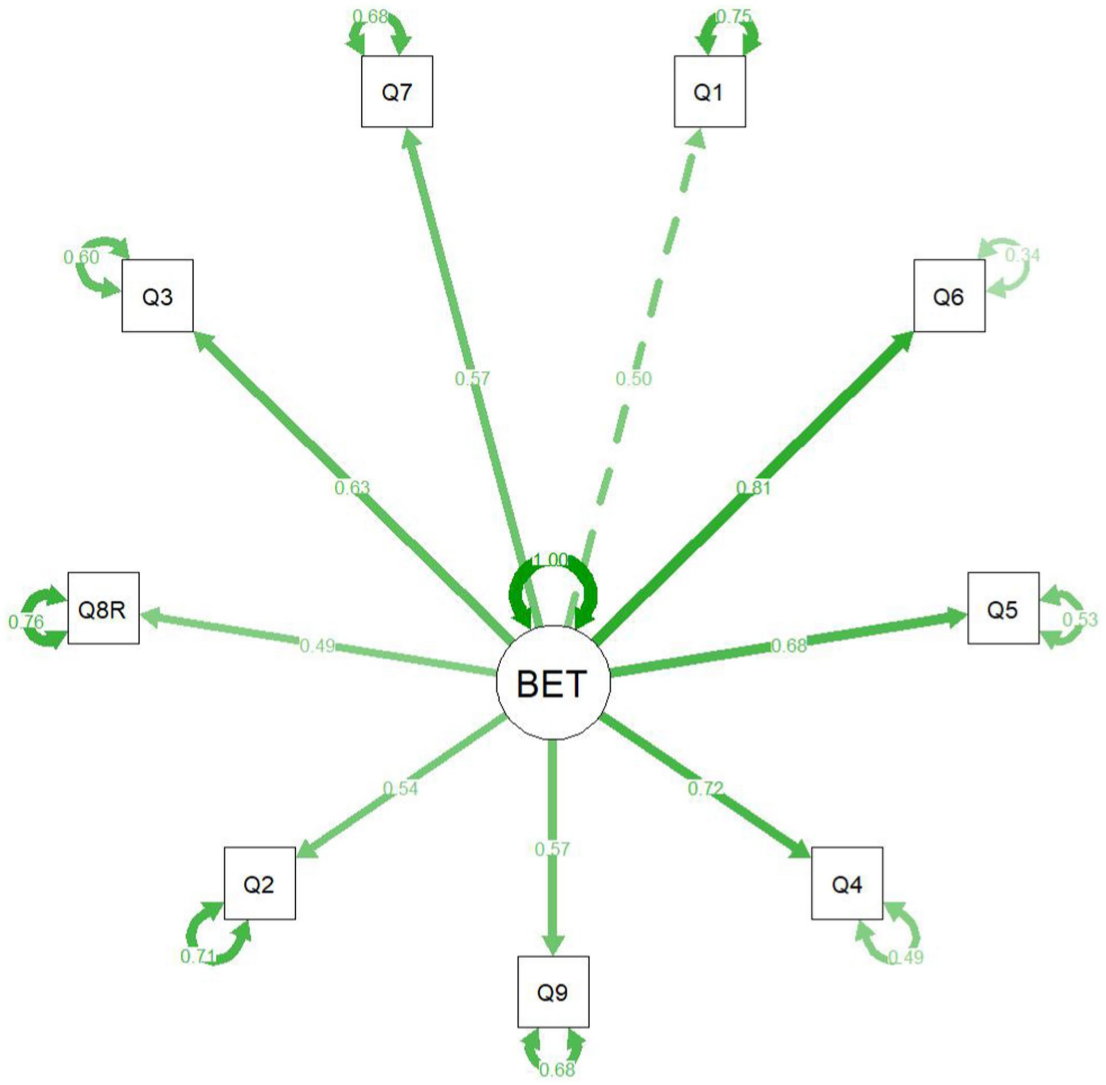
Confirmatory factor analysis plot (study 2).

The confirmatory factor analysis showed an acceptable fit for the one-factor model, CFI = 0.914, TLI = 0.886, RMSEA = 0.094, 95% CI: 0.064, 0.125. Figure 3 presents the factor loading plot for the recommended one-factor solution containing 9 items.

## Discussion

The results of the pilot survey and two consecutive survey waves testing a description of the therapeutic relationship in EMDR therapy as laid out by Hase and Brisch (2022) led to a nine-item solution forming the BET questionnaire, which exhibited acceptable psychometric properties. In addition to the core survey items describing the therapeutic relationship, we also acquired demographics data.

The descriptive items contain the elements of sensitive and predictable behavior, which is considered to be the basis of attachment between infant and caregiver. We assumed that sensitive and predictable therapist behavior could explain the specific therapeutic relationship in EMDR therapy to a great extent. This therapeutic relationship seems to develop rapidly and offer a safe shared space, which allows clients to reprocess, grow, and get past the past. To test this hypothesis, we gathered data by polling therapists trained in EMDR therapy according to an EMDR Europe-based standard, as the basic training requires self-experience in the role of the client. In following, we discuss the results of an internet-based survey eliciting information on the self-experience of therapists during their training in EMDR therapy to evaluate the above-mentioned description of the therapeutic relationship in EMDR therapy.

The demographic data acquired in waves one and two showed that the therapists participating in the survey were mostly in the second half of their professional career. Wave two revealed that the majority (93.3%) of EMDR therapists responding to the survey were between 40 and 79 years of age. This seems to indicate that at least in Germany, therapists begin to practice EMDR Therapy in the middle of their professional career; and in a way continue to practice or remain somehow affiliated to EMDR Therapy, as 41.6% of those who responded to the survey were in the age range between 60 and 79.

Regarding the experience in the role of the client, 30.2% of the surveyed therapists in wave two reported a maximum of 4 sessions in the role of client, which is in accordance with the minimum requirements of an EMDR Europe-accredited EMDR training. Astonishingly, more than two thirds reported more experience in the role of client. This seems to indicate that the therapists sought more self-experience in EMDR therapy than officially required; potentially for other personal benefits. 11.4% reported more than 25 sessions, which could more likely be called therapy.

Regarding the core survey items, the statistical analysis of wave one showed a satisfactory fit for a one-factor model. The factor score adequacy was acceptable with a regression score-factor correlation of 0.88 and a multiple *R*^2^ of scores with factors of 0.77. The reduction of the list of core survey items to 9 items was tested in the second wave. The confirmatory factor analysis showed an acceptable fit for the one-factor model, thus confirming this model in an independent sample exhibiting acceptable psychometric properties. The descriptive statistics for the individual items as well as the entire scale showed good internal consistency.

The descriptive items based on the description of sensitive behavior by Brisch (2002) are supposed to reflect sensitive and predictable behavior by an EMDR therapist asking the client to indicate their agreement on a five-point scale. Items one to six describe the sensitive behavior of the therapist, whereas items seven to nine describe the development and quality of the therapeutic relationship. As the one-factor model was supported by the factor analysis, one can assume that sensitive therapist behavior is closely related to the development of the therapeutic relationship in EMDR therapy.

Even if a sample of psychotherapist seems to be a reasonably representative group regarding mental-health problems compared to the general population, limitations to this study are the relatively small number of participants and the sample that consisted only of therapists trained in EMDR Therapy. Especially one could question if the data and thus the drawn conclusions could be generalized to severly impaired multiply traumatized individuals. This study was conducted with EMDR therapists who perhaps have a higher level of capacity and readiness to engage in a therapeutic relationship. Severly impaired clients come in with a need for varying amounts of stabilization strategies and interventions relevant to their problems in order to maintain dual awareness during EMDR memory processing. Further, clients will differ in the amount of time (and sessions) needed to build up trust in the therapeutic relationship. Hence, this study needs to be viewed within a broader therapeutic framework with EMDR therapy integrating within an overall treatment plan based on assessment of the client and readiness for EMDR memory work. These issues should be addressed in future research. In questioning diverse groups of patients regarding their experience with EMDR Therapy, one could gain additional information more closely related to the client experience. In addition, the list of descriptive items could be used to create a questionnaire measuring the quality of therapist behavior, the development, and quality of the therapeutic relationship in an EMDR Therapy setting.

## Implications for training, consultation, and research

Due to the very short training in EMDR Therapy, it seems understandable that therapists seem to start with EMDR Therapy in the midst of their career, as they first have to undergo extensive training to acquire basic psychotherapeutic knowledge beforehand. As the sample consisted to great extent of German therapists, it might also reflect the special situation in Germany, where EMDR Therapy can only be applied when embedded in one of the major psychotherapeutic approaches; that is, behavioral, systemic, psychodynamic therapy, or in modified psychoanalysis. Regarding the dissemination of EMDR therapy, it is desirable to reach out to therapists early in their career, but this would possibly call for an extension of the EMDR training or integration in university education.

The fact that more than two thirds of the therapists had obviously more experience in the role of client than required in the basic training is remarkable, as this is not mandatory in the current EMDR Training requirements. This could encourage the regulatory bodies like EMDR Europe Association to require additional self-experience as part of the EMDR therapy basic training. As the data seem to support the attachment theory-based concept of the therapeutic relationship with a sensitive behavior of the therapist as a pivotal element, one could discuss the necessity to enhance education in EMDR therapy with a training in sensitive behavior. To our knowledge, this has been successfully implemented in trainings in attachment-based therapy.

## Summary

A description of the therapeutic relationship in EMDR therapy is important in the development of EMDR Therapy as a psychotherapeutic approach. Therefore, Hase and Brisch (2022) described the therapeutic relationship in EMDR Therapy, pointing out parallels between the therapeutic relationship and the development and core features of an attachment-based relationship. We tested descriptions of the elements of sensitive and predictable therapist behavior in therapists who had completed at least a part of the basic training in EMDR therapy. The factor analysis of the collected survey data supported a one-factor model, thus supporting the assumption that sensitive and predictable therapist behavior is related to the development of the therapeutic relationship in EMDR therapy. Limitations to this study are the relatively small number of participants and the sample consisting of therapists trained in EMDR therapy. These issues should be addressed in future research.

Additional data on demographic factors and the amount of self-experience reported by the therapists indicate that most of the therapists begin to train in EMDR Therapy rather late in their professional career and engage in more self-experience than is required by the standards of training. One should consider promoting training in EMDR Therapy within a university context to better reach therapists in the beginning of their professional careers. In addition, the standards of EMDR training could be adapted to the real-life situation by asking for mandatory self-experience in addition to the self-experience in the training courses. Regarding the finding, that a considerable number of therapist engages in self-experience on their own accords we assume, that a mandatory amount of self-experience would be accepted.

In future research, questioning patients on their experience in the EMDR therapy could bring up additional information more closely related to the client experience. In addition, the list of descriptive items could be used to create a questionnaire measuring the quality of therapist behavior, the development, and quality of the therapeutic relationship in an EMDR therapy setting. This could be helpful for the therapist to adjust his or her behavior.

## Data Availability

The raw data supporting the conclusions of this article will be made available by the authors, without undue reservation.
